# Use of immunoglobulin G homeostatic set point and recovery time in plasmapheresis donor safety monitoring: A retrospective observational cohort study

**DOI:** 10.1111/vox.13800

**Published:** 2025-01-29

**Authors:** Janet V. Warner, Michael J. Drinkwater, Gerard J. Chu, Shane Kelly, Jeremy S. McComish

**Affiliations:** ^1^ Pathology and Clinical Governance, Australian Red Cross Lifeblood Brisbane Queensland Australia; ^2^ School of Mathematics and Physics University of Queensland Brisbane Queensland Australia; ^3^ Pathology and Clinical Governance, Australian Red Cross Lifeblood Sydney New South Wales Australia; ^4^ Pathology and Clinical Governance, Australian Red Cross Lifeblood Melbourne Victoria Australia

**Keywords:** homeostatic set point, immunoglobulin G, individual biological variability, plasma donation, plasmapheresis donor

## Abstract

**Background and Objectives:**

Serum immunoglobulin G (IgG) and total protein are used to monitor plasmapheresis donor safety. However, there is a lack of information from large donor cohorts to determine the best use of these measurements.

**Materials and Methods:**

We identified 230,144 plasmapheresis donors making their first donation between 1 July 2020 and 31 March 2024. IgG and total protein were measured prior to the first donation and then annually, following our donor safety monitoring protocol. We considered individuals who had not donated for 12 months to estimate intra‐individual biological variability of IgG. We compared four models to predict which donors would develop IgG < 6 g/L.

**Results:**

The IgG reference interval for the cohort was 7.67–15.6 g/L. IgG declines 5%–11% after the age of 45 years. The intra‐individual biological variability of IgG (5.2%) is small, indicating that there is homeostatic set point for individual IgG. IgG is reduced by plasmapheresis but recovers to recruitment level after 12 weeks. When plasma is donated every 2–3 weeks, mean IgG plateaus 1 g/L below recruitment concentration. IgG at recruitment is the best predictor of which donors will have IgG < 6 g/L after a year of donations. Total protein is a low‐value test in this context.

**Conclusion:**

Plasmapheresis is safe and sustainable for almost every donor, at the 2‐weekly frequency allowed in Australia. The donors most likely to experience unacceptably low IgG are those with very low recruitment IgG levels. These donors could be recommended 12‐week intervals between donations or other donation types.

**Highlights**

- There is a homeostatic set point for serum immunoglobulin G (IgG) that declines by 5%–11% after the age of 45 years. The strongest predictor of IgG < 6 g/L after plasma donation is low IgG concentration at recruitment.
- IgG concentrations in those who donate every 14–21 days can be expected to fall by approximately 1 g/L and plateau within the first year.
- The time taken to restore IgG to the homeostatic set point after plasmapheresis is approximately 12 weeks.

## INTRODUCTION

Plasmapheresis of blood donors for the therapeutic use of plasma or its constituents has been undertaken for more than 7 decades [1, 2, 3, 4]. Today, most donated plasma is converted by industrial fractionation into plasma‐derived medicinal products. Blood services worldwide are continuously engaged in maintaining and improving the supply of donor plasma in the face of increasing healthcare demand for these essential therapeutics [5, 6, 7].

Increasing donation frequency or volume is an approach to improving supply. However, questions about the safe minimum interval between donations, appropriate donation volumes and the relevance of replenishment time of plasma proteins are still of concern to plasma donation services. This is despite the long history of frequent, sometimes high‐volume, plasmapheresis donations without obvious ill effects on donors. While data about the effects of plasmapheresis on donor protein and immunoglobulin concentrations have been published by many authors, the data sets are relatively small, many predate immunoglobulin quantification by automated immunoassays and span a substantial range of donation frequencies and volumes [8, 9, 10, 11, 12, 13, 14, 15].

Adverse events, such as infections, iron depletion or acute cardiovascular or respiratory events, attributable to immunoglobulin or other protein depletion caused by plasma donation, even in individuals who regularly donate more than once per week or have recurrent immunoglobulin G (IgG) concentrations less than 6 g/L, have not been identified in prior publications [5, 16, 17]. While this may be due to a lack of reporting from the plasma donor population, infection due to immunoglobulin deficiency is typically observed in individuals with pathologically sustained low concentrations of immunoglobulins rather than the transient reduction in immunoglobulin concentration characteristic of plasma donors [18]. Total IgG at a single point in time is not considered a sensitive measure of humoral immunity, and if it is low following plasma donation, and/or slow to return to the concentration before plasmapheresis, it may not imply any impairment in ability to increase specific antibody titres rapidly in response to a pathogen. For example, although lower total IgG concentrations were found in plasma donors undergoing different intensities of plasmapheresis, anti‐cytomegalovirus and anti‐tetanus IgG levels, as specific markers of humoral immunity, were not significantly different when compared with non‐donors [14].

It is accepted that total protein (TP) and IgG levels fall during plasmapheresis donation [13, 14, 19, 20]. Early studies suggest that individuals have a homeostatic IgG concentration set point, and that regular donation causes concentrations to fall slightly but still plateau within the population reference interval [14, 19]. Factors that determine the time for an individual's IgG to return to the concentration set point are not known. This is nuanced and therefore hard to study. Also, the clinical relevance of immunoglobulin recovery time may be influenced by other factors such as the nadir of IgG level, the concentration of an individual's set point, as well as the health of other aspects of the innate and adaptive immune system, age and other comorbidities. Age is of particular interest as our population of mature plasma donors is increasing and there is evidence that IgG decreases after about the age of 60 years [21].

Given the homeostatic disturbance that plasma donation might impose, it is also important to note that there is no evidence that repeated plasmapheresis increases the risk of monoclonal gammopathy [22].

In Australia, the minimum and maximum ages for first donation are 18 and 75 years, respectively. Donors may choose to donate either blood or plasma as long as the minimum interval between plasma donations is 14 days, or 3 months between blood donations. Before each donation, donors complete a standardized health questionnaire to ensure that they are fit to donate and have blood pressure and capillary haemoglobin measured. There is no monetary compensation. Collection volume is based on estimated total blood volume (TBV) and does not exceed 16% TBV or 880 mL including anticoagulant. Serum TP, IgG, immunoglobulin A (IgA) and immunoglobulin M (IgM) are measured at the first donation. TP and IgG are then measured annually, irrespective of donation frequency. An exception is applied to existing donors with IgG <6 g/L who are temporarily deferred for 28 days and retested. If a donor's IgG is persistently <6 g/L, they are deferred for 12 months. If IgG is <5 g/L on any occasion, the donor is permanently deferred from apheresis donations.

As a simpler, cheaper and older assay, measurement of serum TP using the biuret method predates donor safety monitoring by IgG immunoassay [3, 8]. Serum TP is a heterogeneous mixture of multiple components, each with its own physiological regulatory pathways. Immunoglobulins constitute about 25% of serum TP and, while annual serum TP measurement remains a European standard for monitoring the safety of plasmapheresis donors [23], there is no evidence that it adds value in a population where IgG is also monitored. In Australia, low TP results from 57 to 60 g/L are managed according to a protocol that allows the supervising medical officer some discretion. However, if the TP is <57 g/L, the donor is deferred until they have obtained independent medical clearance to continue donation, usually from their general practitioner.

There is a recognized need to improve the scientific basis for current plasma donation practices and to inform the setting of standards. A recent knowledge gap analysis has highlighted the need for additional studies of donor health with respect to donation frequency [6].

Predicting and avoiding donor deferral is an important goal for ensuring blood product supply and may have advantages for donor safety. In this article, we use a very large data set of baseline IgG, IgA, IgM and TP concentrations, donation frequency and age to measure intra‐individual biological variability and ‘time to return to baseline’. We then determine the most reliable way to predict low IgG concentrations in plasma donors from these data.

## MATERIALS AND METHODS

### Demographic and assay data

De‐identified age, sex, IgG, IgA, IgM and TP concentrations for apheresis plasma donors at Australian Red Cross Lifeblood from 1 July 2020 to 31 March 2024 were obtained from the organization's database. Consent for the analysis and publication of data of this nature is given at donor recruitment via a standardized donor questionnaire.

Serum IgG, IgA, IgM and TP were measured on the Architect c8000 (Abbott Diagnostics) from 1 July 2020 to 11 August 2023 and the Alinity c (Abbott Diagnostics) thereafter using the manufacturer's validated assays. Assay verification for the Architect c8000 to Alinity c instrument change demonstrated strong concordance, as expected for assays with the same formulation, method (immunoturbidimetry for the immunoglobulins, biuret for TP) and manufacturer. The Australian Red Cross Lifeblood donor testing facility is accredited to AS ISO 15189:2023.

The data were restricted to donors making their first plasma donation (‘new donors’ with ‘recruitment’ testing) during the sample period (230,144 individuals) to provide initial IgG_t1_, IgA_t1_, IgM_t1_ and TP_t1_ measurements for all individuals unaffected by previous plasma donations. While these donors were new to plasmapheresis, they may have donated whole blood in the past. Our standard donor safety testing protocol is to obtain second IgG (IgG_t2_) and TP (TP_t2_) measurements at least 365 days after the first. A total of 173,102 individuals donated plasma for the first time early enough in the data period for a second measurement, but only 60,712 continued to donate plasma long enough for the second measurement. The mean interval between first and second measurements was 514 days (standard deviation [SD] = 180 days). We defined a third sample as donors who donated plasma on the day IgG_t1_ and TP_t1_ were measured but did not donate before IgG_t2_ and TP_t2_ were measured 365 days, or more, later (10,735 individuals). This sample was used to estimate the intra‐individual biological variability of IgG in donors who had not donated plasma for at least 364 days. There were no significant differences in the demographics of these first three samples (Table 1).

**TABLE 1:** Demographics of the plasma donor samples used.

Sample	*N*	Male	Female	Age (years), mean (SD)	IgG_t1_ (g/L), mean (SD)
1. New donors	230,144	105,882	124,262	37 (14)	11.1 (2.2)
2. IgG_t2_ measured	60,712	29,972	30,740	38 (15)	11.0 (2.1)
3. Only one donation before IgG_t2_	10,735	5220	5515	35 (13)	11.1 (2.2)
4. Donations every 14–21 days for 1 year	1128	735	393	46 (16)	10.8 (1.9)
5. Donations every 14–21 days for 2 years	300	220	80	45 (15)	10.8 (1.8)

Abbreviations: IgG_t1_, immunoglobulin G measurement before first donation; IgG_t2_, immunoglobulin G measurement after at least 1 year; SD, standard deviation.

We defined two additional samples of donors who donated every 14–21 days (our minimum donation interval is 14 days) until a second IgG measurement (1128 individuals) and a third IgG measurement (300 individuals). We used these samples to measure the effect of frequent donation on donor health. The demographics of these samples were different from the previous samples with more male donors and a higher mean age (see Table 1).

### Age‐ and sex‐related reference intervals

The refineR software package was used to estimate age‐ and sex‐related IgG reference intervals in the 230,144 new donors. Outlier results were not removed as it is a feature of the software to identify and exclude pathological data. Confidence intervals (95%) were computed from 200 bootstrap iterations [24, 25].

### Intra‐individual biological variability

The difference in IgG measurements, IgG_t2_ − IgG_t1_, was used to estimate the intra‐individual biological variability as follows. We assumed each IgG measurement for a given donor, ‘i’, fluctuates around a homeostatic set point for that donor according to their intra‐individual biological variability CVI_*i*_, and the analytical imprecision (CVA) of the IgG immunoassay [26]. If we then consider the difference in two measurements for the same donor, we define its total variability as CVTD_*i*_. CVI, CVA and CVTD are the SDs of each quantity. Assuming there is no correlation between successive measurements [27], CVTD_*i*_ is given by

$${\text{CVTD}}_{i}=\sqrt{2{\mathrm{CVI}}_{i}^{2}+2{\mathrm{CVA}}^{2}}.$$

If we further assume that the biological variability CVI is the same for all donors, then the total variability in the difference between two measurements for all donors, CVTD, is

$$\text{CVTD}=\sqrt{2{\mathrm{CVI}}^{2}+2{\mathrm{CVA}}^{2}}.$$

We measure CVTD directly from the data. The analytical imprecision, CVA, is calculated from between‐ and within‐run analyser quality control measurements to be 2%. We can then estimate the average intra‐individual biological variability CVI as

$$\mathrm{CVI}=\sqrt{\frac{1}{2}{\mathrm{CVTD}}^{2}-{\mathrm{CVA}}^{2}}.$$

### Time after donation to restore IgG to set point

We estimated the time to restore IgG to the homeostatic set point after plasma donation by calculating IgG_t2_ − IgG_t1_ as a function of the time between IgG_t2_ and the last donation before IgG_t2_. We calculated the average change in IgG in bins of 2 weeks or more since the previous donation, chosen to have at least 1000 donors in each bin. We also split this sample into three groups according to the total number of donations between IgG_t1_ and IgG_t2_ to see whether the recovery was affected by donation rate.

### Prediction of IgG_t2_ < 6 g/L

We used logistic regression to predict ‘IgG_t2_ < 6 g/L’ using any of the following four models: a ‘five‐parameter model’ (combining IgG_t1_, IgA_t1_, IgM_t1_, age and number of donations between IgG_t1_ and IgG_t2_), a ‘three‐parameter model’ (combining IgG_t1_, age and number of donations between IgG_t1_ and IgG_t2_), ‘IgG_t1_ alone’ or ‘TP_t1_ alone’. We tested the input parameters for correlations and excluded TP_t1_ from the five‐ and three‐parameter models because the immunoglobulins are a large component of TP. We used the gflm function in R with the ‘family’ setting specified as ‘binomial’. We tested the output models and did not find any significant non‐linearity or outliers. We used a receiver operating characteristic (ROC) curve to compare the performance of the four different models. We repeated the models using the mean donation interval instead of the number of donations, but there was no significant difference in their performance.

## RESULTS

### IgG population reference intervals at recruitment

**FIGURE 1:**
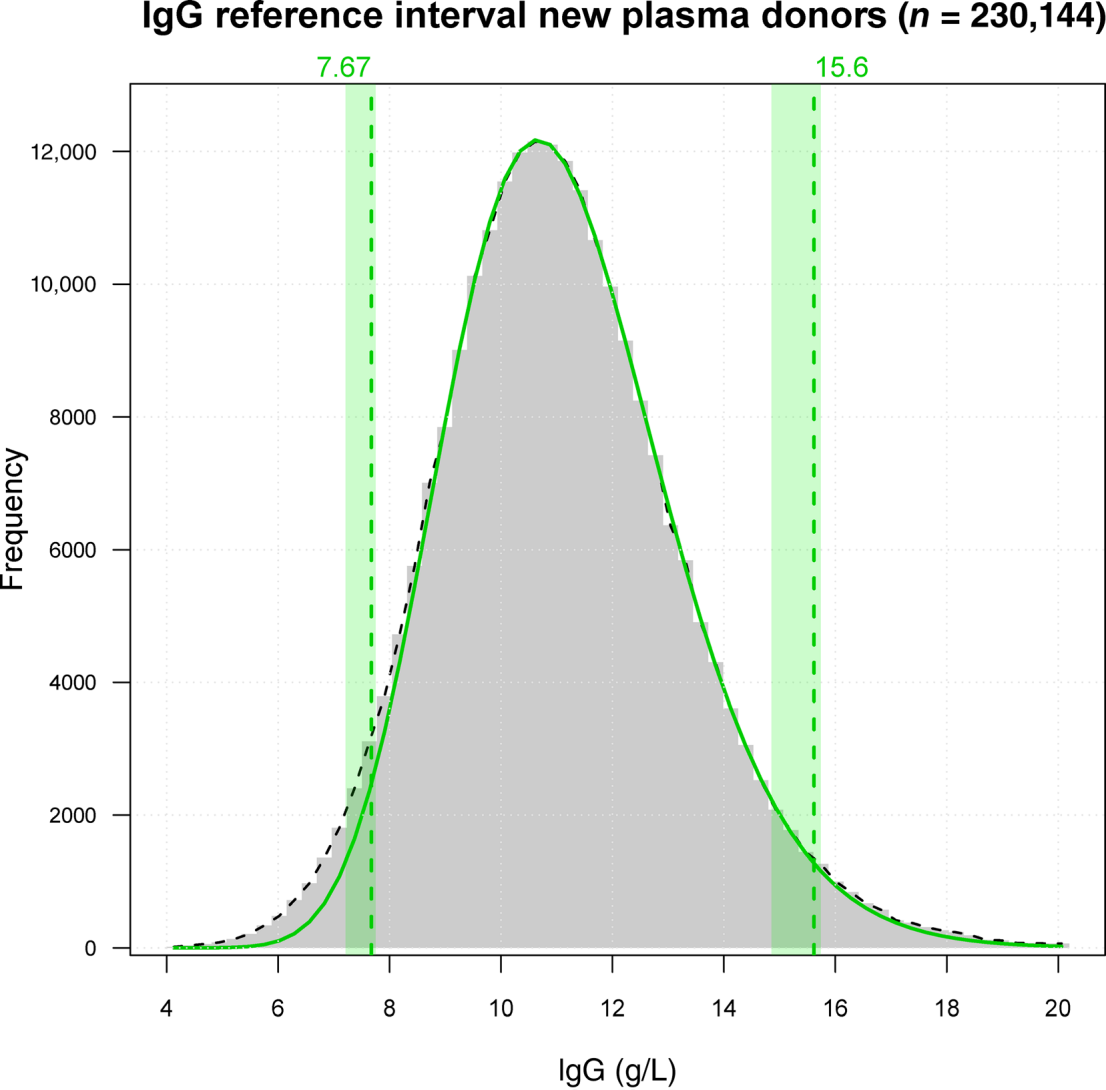
Histogram of immunoglobulin G (IgG) results for new plasma donors (*n* = 230,144) with refineR‐generated upper and lower reference limits as green lines, 95% confidence intervals shaded green.

**FIGURE 2:**
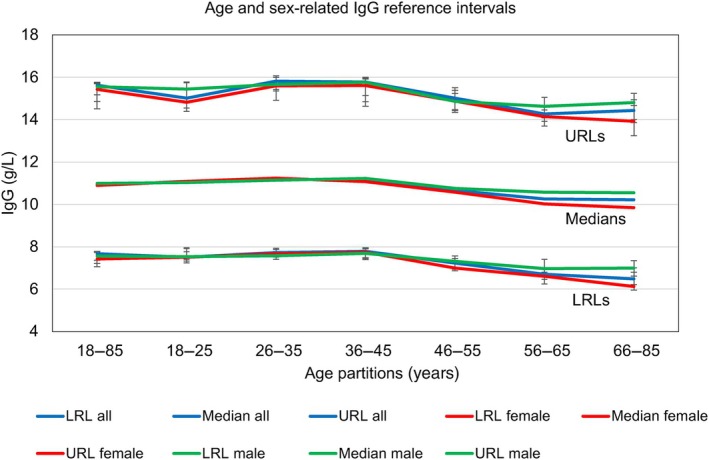
Age‐ and sex‐related immunoglobulin G (IgG) medians and upper (URL) and lower (LRL) reference limits, with 95% confidence intervals, in six age partitions for 230,144 new plasma donors.

Figure 1 shows the distribution and reference intervals of IgG results in 230,144 new donors. Analysis according to age and sex (Figure 2) shows that IgG concentration declines after the age of 45 years by a final average of 5% in males and 11% in females. We calculated the differences by comparing the median levels for ages 66–85 to the medians for ages 18–45.

### Donor age and donation rates

The 230,144 new plasma donors ranged in age from 18 to 85 years, with the distribution skewed to younger donors. The mean age was 37, but the most common age was 18.

There was a wide range in donation rates: 88.5% of donors made 1–10 donations, 10.5% made 11–20 donations, and 1.0% made over 20 donations between IgG_t1_ and IgG_t2_. Most donors did not donate at the maximum allowable rate (every 2 weeks). The mean interval between donations was 215 days (SD = 221 days).

**FIGURE 3:**
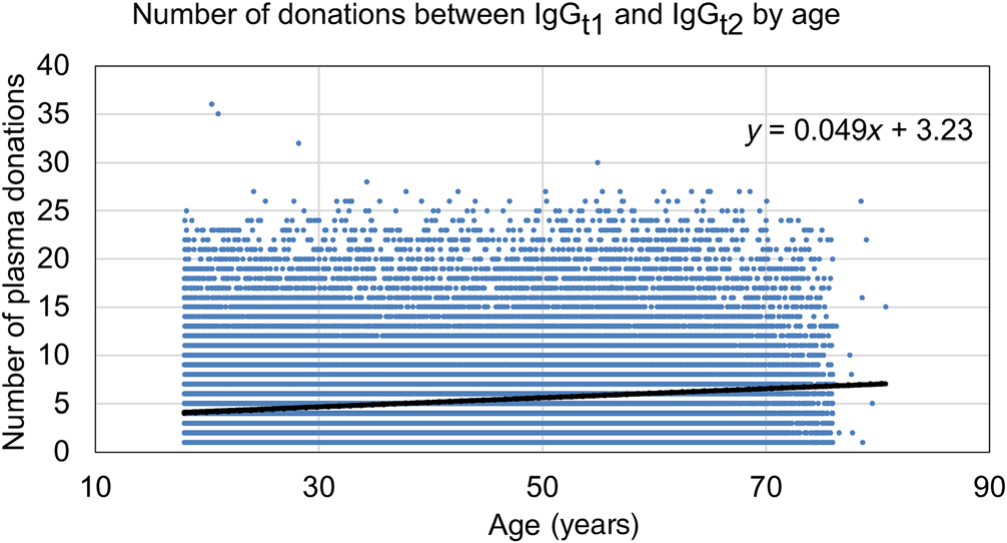
Number of donations between initial (IgG_t1_) and second (IgG_t2_, at least 365 days later) immunoglobulin G measurements, according to age. The dotted line of best fit shows that the number of donations increases with donor age.

There was a significant tendency for older donors to donate more frequently: the line of best fit in Figure 3 (number of donations against age) has a slope of 0.049 (standard error of the mean, SEM = 0.001) donations per year (*p* < 0.001).

### Intra‐individual biological variability and a homeostatic set point

The SD of IgG_t2_ − IgG_t1_ in the 10,735 donors who underwent plasmapheresis on the day of recruitment then not until IgG_t2_ was measured, at least 365 days later, was 0.88 g/L. We calculated the intra‐individual biological variability (CVI) of IgG for this group as 5.2% overall, 5.0% for males (*n* = 5220) and 5.4% (*n* = 5515) for females.

### IgG steady state in regular donors

**FIGURE 4:**
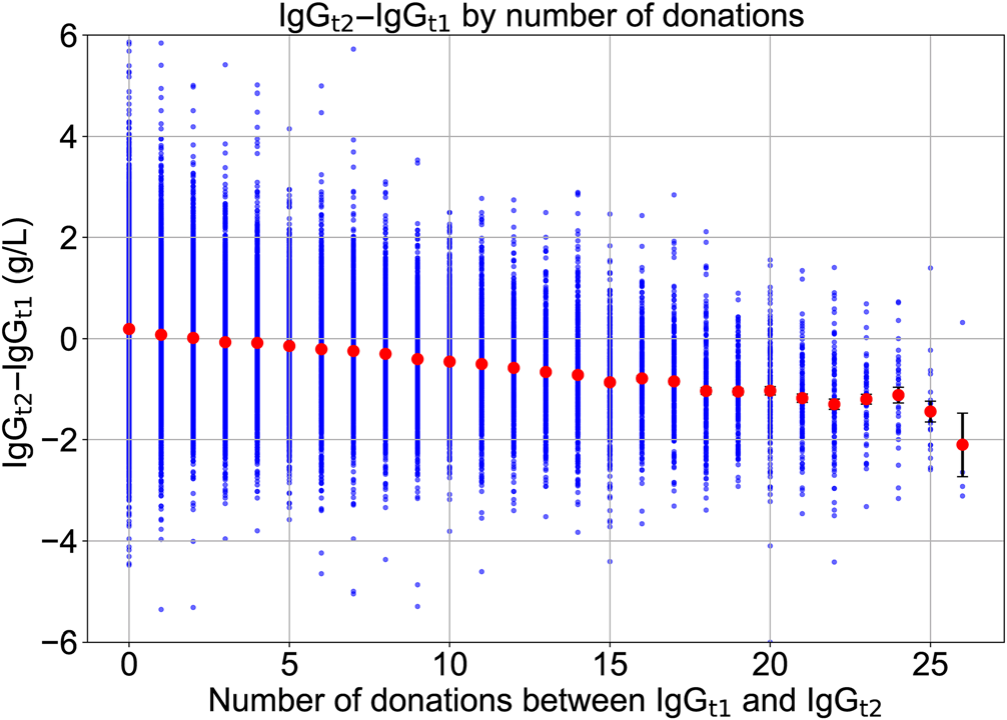
The decline in immunoglobulin G after recruitment as a function of the number of donations in the 12 months before the second measurement (*n* = 60,712). IgG_t1_ and IgG_t2_ are the immunoglobulin G measurements before the first donation and after at least 1 year. Blue dots = data points; red circles and black bars = mean and standard error of IgG_t2_ − IgG_t1_.

The difference between the first and second IgG measurements (IgG_t2_ − IgG_t1_) was negatively correlated with the number of donations between IgG_t2_ and IgG_t1_ (Figure 4).

Of the 13,424 new donors who had IgG measured after both 1 and 2 years, there were 1128 who donated every 14–21 days in their first year, which is at, or close to, our minimum donation interval of 14 days. They had an average IgG of 1.06 g/L below their mean recruitment level of IgG_t1_ = 10.77 g/L (SEM = 0.06 g/L) after 1 year. In the same group of donors, there were 300 donors who continued to donate at the same rate and had a third IgG measurement (IgG_t3_) 2 years after recruitment. Their IgG had not fallen any further (Table 2).

**TABLE 2:** Average change in immunoglobulin G after 1 and 2 years in individuals who donate every 14–21 days.

Time (years)	Average donation interval (days)	Mean ± SEM difference in IgG since recruitment (g/L)	Number of donors
1	18.4	−1.06 ± 0.03	1128
2	18.4	−0.84 ± 0.06	300

Abbreviations: IgG, immunoglobulin G; SEM, standard error of the mean.

### Time after donation to restore IgG to set point

**FIGURE 5:**
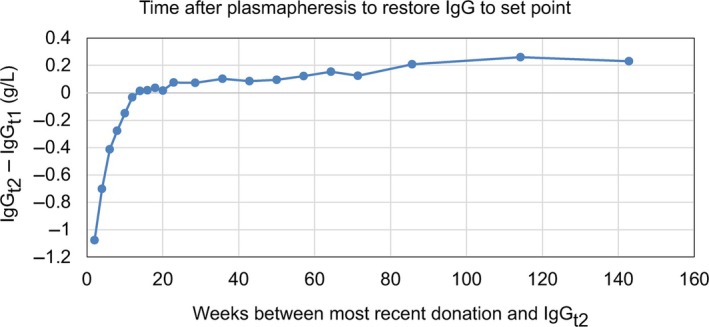
The average change in immunoglobulin G (IgG) as a function of time since the most recent donation. IgG_t1_ and IgG_t2_ are the IgG measurements before the first donation and after at least 1 year. IgG concentration returns to set point an average of 12 weeks post plasmapheresis.

The time taken to restore IgG to the homeostatic set point after plasmapheresis is shown in Figure 5. This average change in IgG was −1.1 g/L after 2 weeks and recovered to the original (IgG_t1_) concentration after 12 weeks. We found that the recovery time was not affected by the total number of donations, although the initial dip in IgG increased with the number of donations.

The IgG assay exhibited a positive calibration drift of up to 0.2 g/L after January 2023, which had a systematic effect of making IgG_t2_ appear higher for individuals with more than 12 months between their last donation and IgG_t2_ measurement. This drift is included in the analytical CV of 2% (used to estimate intra‐individual biological variability) and is within the manufacturer's specifications for the assay.

### Prediction of IgG_t2_ < 6 g/L

The coefficients of the ‘five‐parameter model’ are shown in Table 3. The strongest individual predictive factor for IgG_t2_ < 6 g/L was the value of IgG_t1_. Age and number of donations between IgG_t1_ and IgG_t2_ improve the predictability of IgG_t2_ < 6 g/L, while IgA_t1_ and IgM_t1_ were not significant.

**TABLE 3:** Logistic regression model to predict when the second immunoglobulin G measurement, IgG_t2_ < 6 g/L.

Parameter	Coefficient	Standard error	*p*‐Value
IgG_t1_	−2.671178	0.115367	<0.001
IgA_t1_	−0.120286	0.105941	0.2562
IgM_t1_	−0.092674	0.151943	0.5419
Age (years)	0.015285	0.004807	0.001
Donations	0.055502	0.007346	<0.001

*Note*: The model coefficients, standard errors and significance (*p*‐value) are shown in rows for each parameter used by the model.

Abbreviations: IgA_t1_, immunoglobulin A measurement before the first donation; IgG_t1_, immunoglobulin G measurement before the first donation; IgG_t2_, immunoglobulin G measurement after at least 1 year of donations; IgM_t1_, immunoglobulin M measurement before the first donation.

Parameters with significance *p* < 0.05 were IgG_t1_, the age at recruitment and the number of donations. The coefficient of IgG_t1_ is negative, so higher values of IgG_t1_ make IgG_t2_ < 6 g/L less likely. Conversely, the coefficients of age and number of donations are positive, therefore increasing either of these makes a low IgG_t2_ more likely. However, the coefficient for IgG_t1_ shows that it makes a much larger contribution than age or number of donations.

The three‐parameter model (which excluded IgA_t1_ and IgM_t1_) is indistinguishable from the five‐parameter model on the ROC curve but would be considered a better model because it only considers three parameters.

**FIGURE 6:**
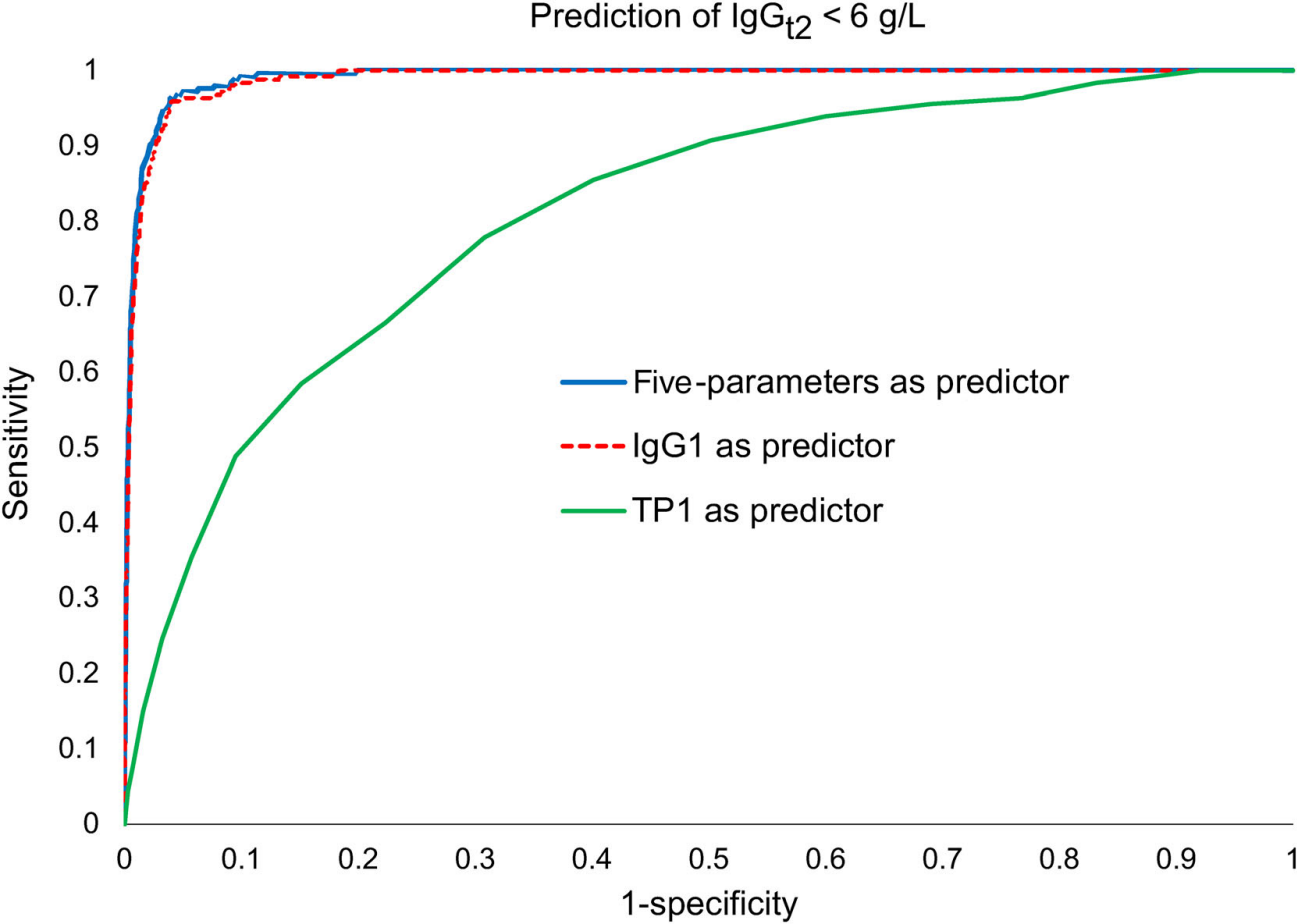
Comparison of three models predicting low second immunoglobulin G measurements (IgG_t2_ < 6 g/L). In this receiver operating characteristic chart, the best models have high values of both sensitivity (the proportion of correctly identified true positives) and specificity (the proportion of correctly identified true negatives), passing close to the top left corner. The ‘five‐parameter’ model (based on the initial immunoglobulins G, M, and A results, age and number of donations) and ‘IgG_t1_ alone’ (based on the initial immunoglobulin G result, IgG_t1_) both perform much better than the ‘TP_t1_ alone’ model (based on the initial total protein result, TP_t1_).

The high dependence and accuracy of IgG_t1_ to predict IgG_t2_ < 6 g/L are illustrated in Figure 6 in which the ROC curve for the five‐parameter model is presented with ROC curves for IgG_t1_ alone and TP_t1_ alone. Using either the five‐parameter model or IgG_t1_ to predict IgG_t2_ < 6 g/L would give very similar results while TP_t1_ is clearly inferior: the maximum Youden indices are 0.924 for the five‐parameter model, 0.914 for IgG_t1_ alone (at IgG_t1_ = 7.8 g/L) and 0.470 for TP_t1_ alone (at TP_t1_ = 69 g/L).

Of our 60,712 donors with IgG_t2_ results, only 248 (0.4%) had IgG_t2_ < 6 g/L. Their IgG_t1_ results are summarized in Table 4 and support the regression results, showing that low IgG_t1_ is a strong predictor of IgG_t2_ < 6 g/L.

**TABLE 4:** Initial immunoglobulin G results of donors whose second immunoglobulin G measurement, IgG_t2_ < 6 g/L.

IgG_t1_ range (g/L)	Number of donors	Number of donors with IgG_t2_ < 6 g/L	Percentage of donors with IgG_t2_ < 6 g/L (%)
5–5.49	34	25	74
5.5–5.99	74	32	43
6–6.49	337	88	26
6.5–6.99	659	60	9
7–7.49	1097	24	2
7.5–7.99	1771	10	0.6
8–8.49	2538	5	0.2
8.5–8.99	3655	2	0.05
9–9.49	4549	2	0.04
≥9.5	45,998	0	0
Total	60,712	248	0.41

Abbreviations: IgG_t1_, immunoglobulin G measurement before first donation; IgG_t2_, immunoglobulin G measurement after at least 1 year of donations.

Those with IgG_t1_ < 6 g/L at recruitment are deferred from future plasma donation until they have been retested and found to have IgG ≥6 g/L.

## DISCUSSION

According to the European Federation of Clinical Chemistry and Laboratory Medicine biological variation database [28], previous estimates of intra‐individual biological variability of IgG range from 1.1% to 6.2%. Our results of 5.2% overall (5% for males, 5.4% for females) are consistent with these earlier studies. While these earlier investigators used more than two IgG results, collected at shorter intervals per individual to estimate intra‐individual biological variability, our sample of 60,712 healthy plasma donors and the interval between individuals' IgG measurements are many times larger than what has been published and is valuable confirmation of the conclusions of much smaller studies.

Our results highlight that the intra‐individual biological variability in IgG is relatively small compared with the population reference interval (Figure 1). This is strong evidence for the existence of an individual homeostatic set point for IgG. The demonstration that IgG in healthy individuals is maintained within a narrow homeostatic set point, declining by only 5%–11% with age, is useful information for the management of plasma donation services.

We have shown that the initial measurement of IgG is a reliable predictor of which donors will have IgG_t2_ < 6 g/L after a year of donations. The prediction is slightly improved if age and number of donations are also included. Also, IgG concentrations in those who donate every 14–21 days can be expected to fall about 1 g/L and reach a steady state. The steady state for our donors is reached within the first year of donating every 14–21 days. There is no further decline after a second year, but we cannot exclude changes over a longer period. We note that these results may not apply in countries allowing more frequent plasma donations than the Australian limit of once every 14 days. For instance, there is evidence that IgG falls more than 1 g/L in very frequent donors [29].

TP measurement is a poor surrogate for IgG and may be useful as a back‐up test, but has limited value if IgG is also being measured. Measuring IgA and IgM at donor recruitment does not assist in predicting low IgG in ongoing donors. However, the initial IgA and IgM results can identify pre‐existing deficiencies in these immunoglobulins and a proportion of paraprotein disease, as well as helping to clarify the significance of low IgG results. The initial IgA measurement is also used to identify IgA‐deficient donors who can donate blood products for IgA‐deficient patients at risk for transfusion reactions.

It can be reliably predicted that donors with a recruitment IgG at or above 7 g/L are unlikely to require intervention or deferral due to IgG below the European Directorate for the Quality of Medicines and HealthCare's ‘Blood Guide’ cut‐off of 6 g/L [23], even if donating every 2 weeks at the older end of the allowable age range (75 years for first‐time donors).

Donors with recruitment IgG closer to the cut‐off (i.e., in the range of 6–7 g/L) may benefit from more frequent IgG monitoring. There is a variation in the frequency of IgG measurement worldwide, with some measuring every 3 months [11] or after every 15 donations [15], or annually (as in Australia). A prospective study of a range of IgG monitoring protocols, with clinical outcomes, would be required to clarify optimal donation and testing frequencies.

In view of the homeostatic set point of IgG, donors with recruitment IgG below 6 g/L may not achieve IgG concentrations appropriate for regular plasma donation despite deferral and retesting. Donors with IgG below the cut‐off on annual monitoring may benefit from extended donation intervals of up to 12 weeks, to ensure enough time for the IgG to return to 6 g/L between donations.

While co‐morbidities can affect an individual's IgG levels, the screening criteria for plasma donation would generally exclude donors in poor health. However, deviation from an expected ‘return to set point’ in returning donors may help to identify individuals with an underlying cause for altered immunoglobulins (such as evolving immunodeficiency or plasma cell dyscrasia) requiring further investigation. Innate and adaptive immune function can also impact serum IgG, but it is not practical to investigate these factors in a large cohort of plasma donors because the required assays are labour‐intensive and poorly standardized.

It should not be assumed that immunoglobulin assays are standardized or harmonized between assay manufacturers. Therefore, the numerical values reported here may not be transferrable to plasma collection services using IgG assays from other in vitro diagnostics manufacturers. Comparability could be investigated by comparing donor population reference intervals estimated with other Total IgG assays to ours, estimated using the Abbott Diagnostics IgG assay.

In conclusion, our large data set of healthy plasma donors demonstrates that there is a homeostatic set point for serum IgG with a small intra‐individual variability (5.2%). IgG concentrations in those who donate every 14–21 days can be expected to fall about 1 g/L and plateau within the first year. They do not fall any further despite continued donation at a similar rate. Analysis of data from jurisdictions where plasmapheresis donation is performed more frequently than every 14 days is needed.

## CONFLICT OF INTEREST STATEMENT

The authors declare no conflicts of interest.

## Data Availability

The data that support the findings of this study are available on request from the corresponding author. The data are not publicly available due to privacy or ethical restrictions.

## References

[1] Grifols‐Lucas JA . Use of plasmapheresis in blood donors. Br Med J. 1952;1:854. 10.1136/bmj.1.4763.854 PMC2023259 14916171

[2] Smolens J , Stokes J , Vogt AB . Human plasmapheresis and its effect on antibodies. J Immunol. 1957;79:434–439. 13491854

[3] Kliman A , Gaydos LA , Schroeder LR , Freireich EJ . Repeated plasmapheresis of blood donors as a source of platelets. Blood. 1961;18:303–309. 14456944

[4] Kliman A , Carbone PP , Gaydos LA , Freireich EJ . Effects of intensive plasmapheresis on normal blood donors. Blood. 1964;23:647–656. 14142503

[5] Purohit M , Berger M , Malhotra R , Simon T . Review and assessment of the donor safety among plasma donors. Transfusion. 2023;63:1230–1240. 37073764 10.1111/trf.17369

[6] Schroyens N , D'aes T , De Buck E , Mikkelsen S , Tiberghien P , van den Hurk K , et al. Safety and protection of plasma donors: a scoping review and evidence gap map. Vox Sang. 2024;119:110–120. 37814964 10.1111/vox.13544

[7] Web Annex A . World Health Organization model list of essential medicines – 23rd list, 2023. The selection and use of essential medicines 2023: Executive summary of the report of the 24th WHO Expert Committee on the Selection and Use of Essential Medicines, 24–28 April 2023. Geneva: World Health Organization; 2023 (WHO/MHP/HPS/EML/2023.02).

[8] Cohen MA , Oberman HA . Safety and long‐term effects of plasmapheresis. Transfusion. 1970;10:58–66. 4191819 10.1111/j.1537-2995.1970.tb00706.x

[9] Shanbrom E , Lundak R , Walford RL . Long‐term plasmapheresis: effects on specific plasma proteins. Transfusion. 1972;12:162–167. 4623688 10.1111/j.1537-2995.1972.tb00003.x

[10] Friedman BA , Schork MA , Mocniak JL , Oberman HA . Short‐term and long‐term effects of plasmapheresis on serum proteins and immunoglobulins. Transfusion. 1975;15:467–472. 53919 10.1046/j.1537-2995.1975.15576082222.x

[11] Wasi S , Santowski T , Murray SA , Perrault RA , Gill P . The Canadian Red Cross plasmapheresis donor safety program: changes in plasma proteins after long‐term plasmapheresis. Vox Sang. 1991;60:82–87. 2031342 10.1111/j.1423-0410.1991.tb00879.x

[12] Ciszewski TS , Ralston S , Acteson D , Wasi S , Strong SJ . Protein levels and plasmapheresis intensity. Transfus Med. 1993;3:59–65. 8038898 10.1111/j.1365-3148.1993.tb00105.x

[13] Lewis SL , Kutvirt SG , Bonner PN , Simon TL . Plasma proteins and lymphocyte phenotypes in long‐term plasma donors. Transfusion. 1994;34:578–585. 8053039 10.1046/j.1537-2995.1994.34794330011.x

[14] Tran‐Mi B , Storch H , Seidel K , Schulzki T , Haubelt H , Anders C , et al. The impact of different intensities of regular donor plasmapheresis on humoral and cellular immunity, red cell and iron metabolism, and cardiovascular risk markers. Vox Sang. 2004;86:189–197. 15078254 10.1111/j.0042-9007.2004.00408.x

[15] Schulzki T , Seidel K , Storch H , Karges H , Kiessig S , Schneider S , et al. A prospective multicentre study on the safety of long‐term intensive plasmapheresis in donors (SIPLA). Vox Sang. 2006;91:162–173. 16907878 10.1111/j.1423-0410.2006.00794.x

[16] Schreiber GB , Becker M , Fransen M , Hershman J , Lenart J , Song G , et al. Plasmavigilance—adverse events among US source plasma donors. Transfusion. 2021;61:2941–2957. 34390267 10.1111/trf.16612 PMC9291118

[17] Moog R , Laitinen T , Taborski U . Safety of plasmapheresis in donors with low IgG levels: results of a prospective, controlled multicentre study. Transfus Med Hemother. 2022;49:271–279. 37969863 10.1159/000522528 PMC10642530

[18] Furst DE . Serum immunoglobulins and risk of infection: how low can you go? Semin Arthritis Rheum. 2009;39:18–29. 18620738 10.1016/j.semarthrit.2008.05.002

[19] Burgin M , Hopkins G , Moore B , Nasser J , Richardson A , Minchinton R . Serum IgG and IgM levels in new and regular long‐term plasmapheresis donors. Med Lab Sci. 1992;49:265–270. 1339930

[20] Laub R , Baurin S , Timmerman D , Branckaert T , Strengers P . Specific protein content of pools of plasma for fractionation from different sources: impact of frequency of donations. Vox Sang. 2010;99:220–231. 20840337 10.1111/j.1423-0410.2010.01345.x PMC3001115

[21] Lock RJ , Unsworth DJ . Immunoglobulins and immunoglobulin subclasses in the elderly. Ann Clin Biochem. 2003;40:143–148. 12662402 10.1258/000456303763046067

[22] Palmer DS , Scalia V , O'Toole J , Welch C , Yi Q , Goldman M . Incidence of gammopathies in long‐term plasmapheresis donors at Canadian Blood Services. Transfusion. 2015;55:1347–1354. 25647184 10.1111/trf.12991

[23] Guide to the Preparation, Use and Quality Assurance of Blood Components. Available from: https://www.edqm.eu/en/blood-guide. Last accessed 1 Oct 2024.

[24] Ammer T , Schützenmeister A , Prokosch HU , Rauh M , Rank CM , Zierk J . refineR: a novel algorithm for reference interval estimation from real‐world data. Sci Rep. 2021;11:16023. 34362961 10.1038/s41598-021-95301-2 PMC8346497

[25] Ammer T , Schützenmeister A , Rank CM , Doyle K . Estimation of reference intervals from routine data using the refineR algorithm—a practical guide. J Appl Lab Med. 2023;8:84–91. 36610416 10.1093/jalm/jfac101

[26] Brescia V , Tampoia M , Cardinali R . Biological variability of serum 25‐hydroxyvitamin D and other biomarkers in healthy subjects. Lab Med. 2013;44:20–24.

[27] Harris EK , Yasaka T . On the calculation of a “reference change” for comparing two consecutive measurements. Clin Chem. 1983;29:25–30. 6848276

[28] Aarsand AK , Fernandez‐Calle P , Webster C , Coskun A , Gonzales‐Lao E , Diaz‐Garzon J , et al. The EFLM Biological Variation Database. Available from: https://biologicalvariation.eu/. Last accessed 1 Oct 2024.

[29] Mortier A , Khoudary J , van Dooslaer de Ten Ryen S , Lannoy C , Benoit N , Antoine N , et al. Effects of plasmapheresis frequency on health status and exercise performance in men: a randomized controlled trial. Vox Sang. 2024;119:134–143. 37997609 10.1111/vox.13569

